# Emerging Issues in Occupational Health Psychology

**DOI:** 10.3390/ijerph182111621

**Published:** 2021-11-05

**Authors:** Jose M. León-Pérez, Mindy K. Shoss, Aristides I. Ferreira, Gabriele Giorgi

**Affiliations:** 1 Cármides Research Group, Department of Social Psychology, Universidad de Sevilla, 41018 Sevilla, Spain; 2 Department of Psychology, University of Central Florida, Psychology Building, 4111 Pictor Lane, Orlando, FL 32816, USA; Mindy.Shoss@ucf.edu; 3 Peter Faber Business School, Australian Catholic University, 22 Main Street, Blacktown, NSW 2148, Australia; 4 Department of Human Resources and Organizational Behavior, ISCTE-Business School, Avda. das Forças Armadas s/n, 1649-026 Lisboa, Portugal; aristides.ferreira@iscte-iul.pt; 5 Department of Psychology, European University of Rome, 190-00163 Roma, Italy; gabriele.giorgi@unier.it

The world of work is changing dramatically due to continuous technological advancements and globalization (the so-called industry 4.0), the health crisis due to the COVID-19 outbreak, and the ongoing global economic crisis, all of which have forced adaptation to new ways of organizing work that have been slowly incorporated during the last few decades (e.g., home working and teleworking), climate change demands, and social movements such as those trying to offer more sustainable alternatives to the current economic model of neoliberalism and the demand-driven manufacturing system, among other factors. Moreover, several studies have shown that workers’ health and well-being can be at higher risk when organizations face relevant changes and economic turbulence [1].

In that sense, after a peer-review process involving international experts, the 21 papers accepted in this Special Issue are reviews and empirical contributions that highlight the emergence of new psychosocial risks for employees’ health and well-being, which are challenging the existing theoretical models and evidence-based practices in Occupational Health Psychology (OHP). 

Undoubtedly, the COVID-19 pandemic has posed some challenges. As usually occurs when an unexpected catastrophe happens, researchers are exploring the short- and long-term consequences on health and well-being, comparing with previous situations or another similar health-related crisis. In this sense, most studies have focused on the negative aspects of the COVID-19 pandemic. For example, Nicola Magnavita and colleagues conceptualize the COVID-19 pandemic crisis as a macro stressor that is negatively affecting the mental health of workers, particularly those in the healthcare sector [2]. Similarly, Martin Sanchez-Gomez and colleagues considered that the COVID-19 pandemic is a traumatic event that is associated with feelings of fear and impaired mental health [3]. Drawing on the Cognitive Activation Theory of Stress [4], they conducted two studies comprising more than 1100 participants in total. Their data supported the idea that the COVID-19 pandemic is associated with intrusive thoughts that keep people in a hyperactivated state (i.e., hyperarousal) which, in turn, diminishes mental health and increases negative emotions such as fear of social activities and being infected. On the other hand, taking a more positive approach, Georgia Libera Finstad and colleagues [5] reviewed previous studies that have explored resilience and growth at work after being exposed to a traumatic event such as the COVID-19 pandemic.

In a similar vein, other studies have focused on the changes in the work design and working procedures that have been triggered by the COVID-19 pandemic, especially regarding remote work or telework. For example, Ward van Zoonen and colleagues explored the factors influencing adjustment to remote work during the beginning of the COVID-19-related lockdown in a sample of 5452 Finnish employees [6]. Their findings identified the interplay between both environmental and contextual factors in predicting adjustment to remote work. Additionally, Carla Estrada-Muñoz and colleagues [7] concluded that the lack of technological skills can endanger a risk of technostress in Chilean teachers.

Another issue that some studies in this Special Issue have covered is the development of scales to measure factors of psychosocial risks at work in particular groups such as the fatigue inventory adapted to coronary artery disease workers by Julija Gecaite-Stonciene and colleagues [8]. Indeed, people with cardiovascular disease have a higher risk of experiencing fatigue at work and, therefore, normative data in this population are needed. Regarding methodological issues, the study conducted by Aristides I. Ferreira and colleagues [9] addresses one important issue in advancing the field of OHP: combining self-report measures with physiological measures. As one of the typical limitations that studies include is the lack of physiological measures compared to self-reported measures, they discuss studies integrating physiological and self-reported measures and offer interesting further research avenues. Particularly, they reviewed the role of biomarkers and hormones in the relationship between presenteeism (another emerging topic in OHP) and performance.

Furthermore, from a methodological point of view, OHP should incorporate more complex designs beyond cross-sectional survey studies to capture dynamic processes in a changing environment. For example, Ieva Urbanaviciute et al. [10] conducted a study with two measurement points in a sample of 959 employees working in Switzerland. They used a latent transition approach to explore dynamics between psychosocial work environment and employee well-being. Additionally, Oliver Weigelt et al. [11] reported the results of a weekly diary study that explores how positive and negative events affect engagement over time. According to theoretical frameworks that advocate for analyzing how certain events can produce behavioral change in organizational contexts [12,13], they focus on the effects that exposure to discrete events at work have on workers’ engagement through the emotions associated with such events. Their results revealed “that positive events accumulate to feed continuously high levels of work engagement over periods of several months” (p. 23). Finally, following a qualitative approach, Isabell Koinig and Sandra Diehl [14] report the key role of healthy leadership and the adoption of workplace health promotion practices, as part of the organizational culture, to develop healthy organizations.

Following a similar positive approach, some authors have explored how personal and social resources can increase employee well-being and performance. For example, Wenqing Tian et al. [15] analyzed some mechanisms that can shed some light on the relationship between job crafting and creativity at the individual level. Additionally, Jean-Sébastien Boudrias et al. [16], in line with the happy-productive worker hypothesis, explore the boundary conditions for the association between employee well-being and proactive performance. Finally, Xiulan Cheng et al. [17] examine how emotional intelligence mediates the relationship between mindfulness and psychological distress in a sample of kindergarten teachers.

In line with the United Nations’ goals for a sustainable and healthy development [18], several studies have analyzed how certain features of individuals, teams, and organizations are associated with more sustainable and productive behaviors in complex environments. For example, Silu Chen et al. [19] reported how more sustainable and green HR practices can promote workers’ green behaviors in industries with high environmental impact; Xiao Deng et al. [20] examined how more dynamic environments are associated with higher entrepreneurial innovation; and Silu Chen et al. [21] investigated how paradoxical leadership (i.e., using strategies that simultaneously balance and satisfy both structural and individual needs) is related to task performance.

Finally, from a stressor–stress–strain perspective, several studies have explored the association of diverse work-related factors with key outcome variables in OHP. Mateo-Rodríguez et al. [22] focused on occupational risk factors for work ability depending on sociodemographic information, which is crucial for identifying exposure risk groups and therefore implementing more accurate and/or tailored measures. Miriam Benítez et al. [23] conceived intragroup conflict as an interpersonal stressor that can negatively affect well-being at the team level and, in turn, decrease the quality of service the unit provides (reported by customers). Gabriela Petereit-Haack et al. [24] conducted a systematic review and meta-analysis of the occupational risks that are related to post-traumatic stress disorder and trauma-related depression. Anne Richter et al. [25], in line with the differentiation between challenge versus hindrance stressors, challenge more traditional stress models and explore how job demands can being associated with positive outcomes. Additionally, Lu et al. [26] revealed the importance of examining the work and family interface by introducing different aspects of gender that capture better nontraditional gender identities.

In sum, the studies included in this Special Issue come from several disciplines and cultural contexts, involving authors from more than 15 different countries. These studies use strong and innovative theoretical approaches to provide evidence regarding the importance of working characteristics and resources to promote healthier and more sustainable environments in which employees can be happy and productive. Moreover, their findings offer several clues for implementing measures and developing healthier organizations in an uncertain and changing environment, particularly in the post-pandemic era. We hope the readers can benefit from the insights of these papers and that their findings can attract the attention of the scientific community in order to pursue further investigation into emerging issues in OHP.

## Data Availability

Not applicable.
